# Does Participation in Local Non-agricultural Employment Improve the Mental Health of Elderly Adults in Rural Areas? Evidence From China

**DOI:** 10.3389/fpubh.2021.746580

**Published:** 2021-10-27

**Authors:** Peng Jia, Jincai Zhuang, Andrea Maria Vaca Lucero, Charles Dwumfour Osei, Juan Li

**Affiliations:** ^1^School of Management, Jiangsu University, Zhenjiang, China; ^2^School of Business, Guilin University of Electronic Technology, Guilin, China

**Keywords:** non-agricultural employment, mental health, elderly adults, rural areas, China

## Abstract

A rising rate of suicide among the elderly in rural China has been recognized to be triggered by mental health-associated factors. This study uses 3,397 sampled rural elderly adults from China Labor-force Dynamic Survey in 2016 to explore the response mechanism through which non-agricultural employment participation by the elderly adults in rural China can influence their mental health. Utilizing the Multivariate Regression, Instrumental Variable and Propensity Score Matching methods, we find that, the rural elderly adults who participate in local non-agricultural employment significantly improve their mental health. Self-employment tends to have a greater positive contribution to the mental health of the elderly population than waged employment. Further, work income, need for belongingness and respect, and human capital development significantly mediates the influence of participation in local non-agricultural employment on the mental health of the elderly adults. Finally, we put forward relevant policy suggestions to improving the mental health of the elderly in the countryside.

## Introduction

In the context of the increasing situation of rural aging in China, the pension and health problems of rural elderly adults need to be concerned. According to the official data of the latest national population census, there are 264 million people over 60 years old in China, representing 18.7% of the total population, nearly twice as high as the international standard for defining an aging society (10%). Moreover, in the process of China's industrialization and urbanization, a considerable number of young people from rural areas move to the cities (1, 2), while the elderly adults over 50 years old are left behind due to age and educational barriers (3). From the perspective of this reality, the outflow of young people from rural areas may harm the health of the left-behind elderly adults (4). Young people going out for employment opportunities will bring intergenerational residential separation. Making it difficult to meet the daily care and inner comfort of the left-behind elderly adults in rural areas (5). Furthermore, the outflow of the young labor force leads to the lack of endogenous power in rural economic and social development, while the infrastructure and public services related to medical care and pension in villages lag far behind (6). As a result, the health conditions of the rural left-behind elderly adults are difficult to be guaranteed.

Mental health represents a critical aspect of elderly adults' well-being. The gradual deterioration of mental health will accelerate the potential loss of daily behavior ability (7). In recent years, the suicide rate of the elderly in rural China continues rising (8), and the deterioration of mental health is one of the trigger factors. Mental health deterioration is mainly manifested in losing confidence in life and believing that life is meaningless (9, 10). For a long time, the mental health of elderly adults in rural areas has been of interest to scholars, mainly focusing on the influencing factors like family population mobility (11–13), medical and social endowment insurance (14–16), widowhood, and disability (17–19), and so on. It is not difficult to find that most of the present studies are related to the perspective of “negative aging” to explore how to improve the mental health of rural elderly adults. However, life expectancy has increased at present, and many older adults are still active and able to continue to engage in labor even when they reach retirement age (20). Moreover, the growth of individual age will not decrease experience and wisdom (21), and these attributes represent a valuable wealth for rural economic and social development. According to the theory of active aging, improving active social participation is a fundamental key to help the mental health of elderly adults (22), and non-agricultural employment is one of the relevant forms to foment active social participation. For this reason, it is of considerable importance to fully tap the rural elderly human capital. Hence, the present study specifically explores the impact of rural elderly adults' participation in local self-employment or waged employment in non-agricultural business on their mental health. The study further examines the channels through which rural non-agricultural employment participation of the elderly contributes to their mental health.

This study employs the national representative data of the China Labor-force Dynamic Survey in 2016 to analyze the impact of rural elderly adults' participation in local non-agricultural employment on their mental health and its mechanism. To begin with, the Ordinary least squares (OLS) model and Ordered logit (Ologit) model are used for the benchmark regression analysis of the data. The instrumental variable method also addresses the endogenous problem, while the Propensity Score Method further tests the robustness of benchmark regression results. In this study, the mediating effect model is used to test the specific path of the impact on the mental health of rural elderly adults participating in local non-agricultural employment.

Compared with previous researches, this study presents the following innovations. Firstly, based on the perspective of “active aging,” this study empirically analyzes the improvement effect of participation in local non-agricultural employment of rural elderly adults on their mental health, which expands the research on rural pension mode. Secondly, the present study innovatively utilizes the instrumental variable method and the PSM method based on quasi-natural experiment ideas to test the endogeneity of the benchmark model and produce robust results. Thirdly, this study tests the specific path of rural elderly adults' participation in local non-agricultural employment on their mental health, and reveals the influence mechanism of local non-agricultural employment on rural elderly adults' mental health, which is of great significance to solve the rural public health problems.

The ensuing contents of this paper are arranged as follows: The second part is the theoretical foundation and research hypotheses. The data and methodology are presented in the third part. The fourth part is the empirical results and discussion, while the fifth part covers the conclusions and policy recommendations.

## Theoretical Foundation and Research Hypotheses

Role theory is one of the frequently used theories to explain the mental health changes of elderly adults. Role theory assumes that a person's life is a stage, and each scene on the stage changes with age, and each role's change will affect the individual's psychological state (23). The elderly adults in rural China are experiencing three main changes in their aging process:

Firstly, the change from an active working mode to a leisure role. Most rural people actively work in cities while young. However, once entering the elderly adult stage, due to the limitations of physical conditions and lack of updated skills, the return to their rural homes has become the helpless choice of migrant workers (24). Moreover, due to technology progressively replacing labor, elderly farmers in rural areas do not need to invest too much energy in agricultural production (25). This change from a fast-paced, competitive work-life to a slow-paced, leisurely family life may cause emotional and psychological disorders such as feelings of loss and frustration.

Additionally, the change from a leading position to the dependent role. Elderly adults in rural areas were the primary contributors to their family's income when they were young, and they also held an advantage in social relationships and played a leadership role in their families (26). However, after entering the elderly adult stage, their adult children gradually take part as contributors to their family's income. Thus, the role of parents changed from a family leading position to a dependent position (27, 28). These sorts of changes cause tension in family relationships, having an implicit negative impact on the mental health of elderly adults.

Finally, the change from social protagonist to play a solitaire role. After an individual enters the elderly adult stage, the social relationship starts shrinking, and the role tends to become solo. Due to age restrictions, the rural elderly adults slowly withdraw from the labor market. The multiple functions they play in society get gradually lost, such as their frequency of communication and social activity participation. These changes will make the rural elderly adults feel lonely and empty, causing negative impacts on their mental health (29).

Theoretically, active participation in local non-agricultural employment can improve the mental health of the elderly adults in rural areas from three key aspects: Firstly, participation in local non-agricultural employment can bring sustained and stable income to the rural elderly adults, favoring their economy, alleviating the tension of family relationships, so as to improve their mental health (30). The association between income status and mental health has been reported in the literature. Existing studies reveal that, income status obtained by individuals has a significant influence on their health outcomes, including their mental and physical health, particularly in the case of elderly adults (31–34). Further, findings from (35) reveal that, the likelihood of depressions, mental disorders, and anxiety decrease when economic status or income level rise. Families which higher income quintiles are likely to have low levels of anxiety, depression, and mental health disorders. This underscores the mechanism of the interrelationship between employment participation, level of income, and mental health.

Again, participation in local non-agricultural employment could ensure the continuation of their former professional role, alleviating the mental stress caused by the change of roles, also helping to develop their human capital and acknowledge their value, and so improving their mental health (36, 37). The relationships and pathways between human capital development and physical and mental health have been explored in the literature (8). Improvement of human capital development contributes positively to employment participation through which employees earn incomes and improve their well-being. Developing human capital skills, abilities, and physical strength, contribute to good mental health. Findings from literature (9) suggest that, human capital development has the likelihood to improve productivity, life expectancy, quality of life and physical health, and mental health.

Lastly, participation in local non-agricultural employment can help rural elderly adults to keep socially active, fostering friendly relationships. This may arise from professional affairs, meeting their needs of belongingness and respect, and thus keep enhancing their mental health (38, 39). In this view, the needs for belongingness and respect are particularly important among elderly adults (40, 41). The elderly adults require more social interaction, respect, and acceptance to be emotionally and mentally healthy. According to (41, 42), the sense of belongingness and respect and good interpersonal relationships are basic psychological needs that improve the mental health of the aged population. In this case, when the needs of belongingness and respect are not achieved, it may result in the loss of happiness, rejection, and pain. When these happen, there is a high tendency of such individuals to suffer from the mental problems (42, 43). Previous authors conclude that, to improve the mental health of elderly adults, in particular, their interpersonal relationship, sense of belonging, and respect for such people should be improved either through interaction with people at the workplace or at home (44–46).

In the same vein, the heterogeneity of non-agricultural employment types may have different effects on the mental health of rural elderly adults. Some studies have drawn the conclusions based on self-employment and working as employees (47). Some scholars suggest that, self-employment favors the elderly adults more than waged employment since probably, many employers may be unwilling to employ old aged people who may have lower productivity. Hiring elderly adults will also require high insurance and labor costs, yet they are mostly described as inefficient. There is an argument that, elderly employees mostly have less updated skills and are slow to learn new things than young workers. Hence it is more attractive for elderly adults retiring to venture into self-employment instead of waged employment (48). The elderly adults who remain in the labor market, particularly the non-agricultural employment, often feel more comfortable when they work as self-employed. As corroborated by previous studies, self-employed elderly adults enjoy more life satisfaction than waged work after reaching their retirement age (49). Self-employment offers more motivation for elderly adults and increases their life satisfaction, well-being, and mental health (48). Therefore, the mental health of the rural elderly adults self-employed in non-agricultural activities may be greater than that of the employed group.

Based on the preceding discussion, the following hypotheses are proposed:

**Hypothesis 1:** Participation in local non-agricultural employment has a significant positive influence on the mental health of rural elderly adults.
**Hypothesis 2:** Participation in self-employed non-agricultural work has a significantly greater positive influence on the mental health of rural elderly adults than working as an employee in non-agricultural work.
**Hypothesis 3a:** Participation in local non-agricultural employment has a significant positive influence on the mental health of rural elderly adults through the mediating role of income effect from employment.
**Hypothesis 3b:** Participation in local non-agricultural employment has a significant positive influence on the mental health of rural elderly adults through mediating role of development of human capital exertion.
**Hypothesis 3c:** Participation in local non-agricultural employment has a significant positive influence on the mental health of rural elderly adults through the mediating role satisfaction of belongingness and respect.

## Data and Methodology

### Data

#### Data Sources

The empirical data of this study comes from the 2016 China Labor-force Dynamic Survey (CLDS_2016). CLDS is a large-scale micro survey project organized and implemented by Sun Yat-sen University, covering many research topics such as education, work, migration, health, social participation, economic activities, and grassroots organizations. It is an interdisciplinary large-scale follow-up survey. CLDS adopts the multi-stage, multi-level, and proportional probability sampling method. The data sample covers 29 provinces in China (excluding Hong Kong, Macao, Taiwan, Tibet, and Hainan), and the sample is generally representative of China (50).

Since this study mainly focuses on the impact of rural elderly adults' participation in local non-agricultural employment on their mental health, the following exclusion and inclusion activities were undertaken in the sample selection procedure: (a) All the urban samples were deleted while retaining only the rural samples. (b) The respondents' household registration was limited to rural areas, and the age ranges from 50 to 80 years were considered. (c) The samples without jobs were excluded, and the working place of the respondents was limited to the county area where they currently lived, including villages, towns in the counties. (d) The samples with serious missing data were deleted. Through these activities, 3,397 valid samples were obtained.

#### Description of Main Variables

Dependent variable: The mental health condition of elderly adults in rural areas. Referring to the research of Yang and Jiang (51), this study uses the CLDS_2016 individual questionnaire, inquiring “how often do you feel that life is meaningless?,” the statement is measured with a four-point Likert scale (1 = Almost always, 4 = Rarely). Generally, the more individuals feel that life is meaningless, the higher the tendency to deteriorate mental health. Therefore, we re-encoded the data based on the four-point Likert Scale (1 = Poor, 4 = Very good).

Independent variable: Local non-agricultural employment. The construction of this variable comes from the CLDS_2016 individual questionnaire item: “Are you working as an employee, employer, self-employed, or in farming?,” According to the definition of the four types of employment in the CLDS questionnaire, this study defines employee, employer and self-employed as non-agricultural employment, giving a value of 1 to these three types, and a value of 0 to farming. Note that the respondents' scope of work has been limited to the county district where they currently lived in the sample selection process, reducing the description of “local” on this part.

Mediating variables: Based on the second part of the theoretical analysis, this study constructs three mediating variables: needs of belongingness and respect, income effect, and human capital development, to investigate the mechanism of rural elderly adults' participation in local non-agricultural employment on their mental health. Based on the CLDS_2016, the “needs of belongingness and respect” variable is measured by the subjective evaluation of the respondents on the satisfaction of being respected in their current work, using a five-point type Likert scale response, ranging from 1 = not satisfied at all to 5 = extremely satisfied. The variable of “income effect” was measured by the item “your satisfaction with the current work income,” using the same five-point Likert scale. The variable of “human capital development” was represented by the satisfaction degree of the interviewees to their ability and skills, and was also measured by the same five-point Likert scale.

Control variables: Referring to the existing studies (38, 52), this research divides the factors that may affect the health of the elderly adults in rural areas into four categories, including individual characteristics, family characteristics, village characteristics, and province marks. The individual characteristics of the interviewees include gender, age, marriage, education, and previous health condition. The family characteristics include new rural insurance and numbers of families living together. The village characteristics include the distance between villages and towns, local social support, and village environment. The province indicator is assigned from 1 to 29, which is used as a regional fixed effect in the econometric regression model.

#### Descriptive Statistics

The results from the descriptive statistics on the outcome variables utilized in the study are reported in Table 1. Overall, Table 1 indicates that about 78.83% of the rural elderly adults maintain very good mental health. Regarding independent variables, the results show that 20.16% of the respondents participate in non-agricultural employment, while 79.84% are engaged in farming. Moreover, out of the total sampled respondents, 448 participate in waged employment accounting for 14.18% while 237 people participate in self-employed work, accounting for 8.04%. In terms of mediating variables, about 6.78% are extremely satisfied with their income, while 8.01% are not satisfied at all. The average satisfaction of “Needs of belongingness and respect” is high, of which around 13.41% are extremely satisfied, and 45.08% are very satisfied. The average satisfaction of “Human capital development” is also high, of which around 10.96% are extremely satisfied, however a significant proportion of about 41.57% are very satisfied. In terms of instrumental variable, rural areas with non-agricultural economies account for about 21.34%. In terms of other features in our sample, males account for 56.5%, and the average age of the respondents is about 59 years old. Among the elderly, the majority belong to the married group, and their educational level is generally low. About 12.1% of elderly adults have a bad previous health condition, and 61.94% have New rural insurance. The average number of families living together is 4.892; the average distance between villages and towns is 5.619. Similarly, the average number of local people who can provide support is 10.233 while the village's environmental level is high, with an average of 7.271.

**Table 1:** Descriptive statistical results of variables.

**Variables**	**Observations**	**Mean/%**	**Variables**	**Observations**	**Mean/%**	**Standard error**
**Dependent variable**			*Extremely satisfied*	310	10.96%	
Mental health	3,397		*Very satisfied*	1,176	41.57%	
*Poor*	64	1.88%	*Quite satisfied*	1,086	38.39%	
*Fair*	123	3.62%	*Not very satisfied*	199	7.03%	
*Good*	532	15.66%	*Not satisfied at all*	58	2.05%	
*Very good*	2,678	78.83%	**Instrumental variable**			
**Independent variables**			Non-agricultural economy (*Yes= 1; None = 0*)	3,364	21.55%	
Non-agricultural employment (*Non-agricultural employment = 1; Farming = 0*)	3,397	20.16%	**Control variables**			
Employed (*Employed = 1; Farming = 0*)	3,160	14.18%	Gender (*Male = 1; Female = 0*)	3,397	56.49%	
Self-employed (*Self-employed = 1; Farming = 0*)	2,949	8.04%	Age	3,397	58.952	6.635
**Mediating variables**			Marriage (*Married = 1; Others = 0*)	3397	94.52%	
Income effect	3,332		Education	3,397		
*Extremely satisfied*	226	6.78%	*Primary school and below*	2,079	61.20%	
*Very satisfied*	1,120	33.61%	*Junior middle school*	1,013	29.82%	
*Quite satisfied*	1,026	30.79%	*High school*	293	8.63%	
*Not very satisfied*	693	20.80%	*Undergraduate and above*	12	0.35%	
*Not satisfied at all*	267	8.01%	Previous health (Bad *= 1; Good = 0*)	3,397	12.10%	
Needs of belongingness and respect	2,491		New rural insurance (Yes *= 1; No = 0*)	3,397	61.94%	
*Extremely satisfied*	334	13.41%	Number of families living together	3397	4.892	2.283
*Very satisfied*	1,123	45.08%	Distance between villages and towns	3,397	5.619	5.161
*Quite satisfied*	856	34.36%	Local social support (*numbers of local people who can provide support*)	3,397	10.233	23.430
*Not very satisfied*	143	5.74%	Village environment (*ranging from 1 = very messy to 10 = very neat*)	3,397	7.271	1.642
*Not satisfied at all*	35	1.41%	Province marks (*different provinces are assigned 1–29 in turn*)	3,397	–	–
Human capital development	2,829					

### Methodology

#### Benchmark Model

Based on the previous theoretical analysis, to test the impact of rural elderly adults' participation in local non-agricultural employment on their mental health, this study constructs the following benchmark model:


(1) $$\begin{array}{r} Health_{i}=\alpha_{0}+\alpha_{1}Employ_{i}+\sum \gamma_{m}X_{mi}+\phi Province_{j}+\varepsilon_{i} \end{array}$$

Where *Health*_*i*_ is the mental health variable of the interviewees; *Employ*_*i*_ is the core explanatory variable, to explain the participation of elderly adults in local non-agricultural employment in rural areas, being a binary dummy variable, where the value of participating in local non-agricultural employment is 1, otherwise, it is 0. *X*_*mi*_ is a series of control variables that may affect the dependent variables, including the individual, family, and village characteristics of the respondents; *Province*_*j*_ is the symbol of province; ε_*i*_ is the random error term.

The dependent variable in this paper is an ordered category variable. Therefore, we mainly select the Ordered logit (Ologit) model for regression analysis, and take the OLS model as a reference. In using the Ologit model, we assume that ε_*i*_ follows the logistic distribution.

#### Mediating Effect Model

To identify potential mediating effects, related to practices of existing studies (53), this paper constructs the following mediating effect model based on model (1):


(2) $$\begin{array}{r} Mediating_{i}=\alpha_{0}+\alpha_{2}Employ_{i}+\sum \gamma_{m}X_{mi}\\ \;+\phi Province_{j}+\varepsilon_{i} \end{array}$$

(3) $$\begin{array}{r} Health_{i}=\alpha_{0}+\alpha_{3}Employ_{i}+\sum \beta_{3k}Mediating_{ki}\\ \;+\sum \gamma_{m}X_{mi}+\phi Province_{j}+\varepsilon_{i} \end{array}$$

Where *Mediating*_*i*_ represents the intermediary variable. According to the previous analysis, this paper includes the income effect, needs of belongingness and respect, and human capital development as the intermediary variables. The other variables' meaning is consistent with the explanation of model (1). The three mediating variables in this paper are ordered category variables. Therefore, we can use the Ologit model for regression analysis, assuming that ε_*i*_ follows the logistic distribution.

According to the existing studies (53), there are three steps to identify the mediating effect: The first step is to test the benchmark model (1), if α_1_is significant, enter the second step, otherwise, the mediating effect does not exist; The second step is to test the models (2) and (3). If both α_2_ and β_3*k*_ are all significant, the mediating effect exists. If at least one of them is not significant, it needs to be further identified by the Sobel test. Passing the Sobel test indicates that the mediating effect exists. If it fails to pass the test, there is no mediating effect. Sobel test formula is as follows:


(4) $$\begin{array}{r} z=\frac{\alpha_{2}\beta_{3k}}{\sqrt{{\alpha_{2}}^{2}{S_{\beta }}^{2}+{\beta_{3k}}^{2}{S_{\alpha }}^{2}}} \end{array}$$

Where *S*_α_ and *S*_β_ are the standard deviations of the estimated values of parameters α_2_ and β_3*k*_, respectively.

#### Endogenous Test

The study of the influence of local non-agricultural employment on the mental health of elderly adults in rural areas presents endogenous problems due to: (a) Unobservable or missing variables. The mental health of elderly adults in rural areas may also be affected by factors such as character and daily living habits, which are difficult to measure by specific variables; (b) Two-way causal relationship. The health condition may have a reverse effect on the non-agricultural employment decision-making of rural elderly adults. Generally, people in good health are more likely to participate in non-agricultural employment. To solve the endogenous problem, the previous health condition variable is added into the control variables to alleviate possible problems from model 1. In addition, the instrumental variable method has been proved to be an excellent way to deal with endogenous problems in many empirical studies. Hence, this method is also applied to deal with these potential issues in this study.

The choice of instrumental variables should meet two basic conditions: First of all, be highly correlated with endogenous explanatory variables, and instrumental variables have no direct impact on dependent variables (54). Based on this, the study selects a local non-agricultural economy (secondary and tertiary industries) as the instrumental variable for local non-agricultural employment. Its rationality lies in: (a) The local non-agricultural economy can provide conditions for the rural elderly adults to participate in employment opportunities, which meets the assumption that the endogenous explanatory variables are highly related; (b) From the actual situation in rural China and existing researches, there is not enough evidence that the local non-agricultural economy will have a direct impact on the mental health of the rural elderly. Due to age and other reasons, the rural elderly do not necessarily participate in local non-agricultural economic activities. Hence, it will not directly impact their mental health, which meets the exogenous hypothesis of efficient instrumental variables.

#### Robustness Test

In addition to using the instrumental variable method to deal with endogenous problems, this study also uses the propensity score matching method (PSM) to test the robustness of model 1, to further verify the impact of participating in local non-agricultural employment on the mental health of rural elderly adults. The PSM method finds similar samples to the treatment group (participating in local non-agricultural employment) from the control group (not participating in local non-agricultural employment) through the counterfactual analysis framework to construct the counterfactual state of the treatment group corresponding to the control group samples (55). Finally, the average treatment effect on the treated (ATT) of the influence of local non-agricultural employment on mental health was obtained. In addition, considering the estimation of propensity score may still have errors, this study refers to the practice of Zhao and Li (56), and adopts three matching methods, namely k-nearest neighbor matching, radius matching, and kernel function matching, to ensure the robustness of the calculation results of the PSM method.

## Empirical Results and Discussion

### Analysis of Basic Results

Table 2 reports the estimated results of the impact of rural elderly adults' participation in local non-agricultural employment on their mental health. In order to observe the robustness of regression results, the OLS model, and Ologit model are used in this study. Model 1 is the OLS regression result of the core explanatory variable to the dependent variable. Model 2 is the OLS regression result of adding a series of control variables on the basis of Model 1. Model 3 is the Ologit regression result without control variables, and Model 4 adds control variables based on Model 3. It can be seen in Table 2 that the estimated coefficients of local non-agricultural employment variables are all positive, and they all reject the null hypothesis at the statistical level of 1%, indicating that after controlling the respondents' individual characteristics, family attributes, village features, and province marks, the participation of rural elderly adults in local non-agricultural employment can significantly improve their mental health, and hypothesis 1 is supported.

**Table 2:** Basic regression results.

**Variable name**	**Mental health**			
	**Model 1 (OLS)**	**Model 2 (OLS)**	**Model 3 (Ologit)**	**Model 4 (Ologit)**
Local non-agricultural employment	0.153^***^	0.121^***^	0.728^***^	0.590^***^
(Local non-agricultural employment = 1; Farming = 0)	(0.02)	(0.02)	(0.12)	(0.13)
Gender		0.058^**^		0.240^***^
		(0.02)		(0.09)
Age		0.002		0.009
		(0.00)		(0.01)
Marriage		0.015		0.110
		(0.05)		(0.18)
Education		0.040^**^		0.220^***^
		(0.02)		(0.08)
Previous health		−0.220^***^		−0.763^***^
		(0.04)		(0.11)
New rural insurance		0.009		−0.026
		(0.02)		(0.09)
Number of families living together		0.001		−0.002
		(0.00)		(0.02)
Distance between villages and towns		−0.005^**^		−0.024^***^
		(0.00)		(0.01)
Local social support		0.001^***^		0.006^**^
		(0.00)		(0.00)
Village environment		0.000		0.011
		(0.01)		(0.03)
Province mark	No	Yes	No	Yes
Observations	3,397	3,397	3,397	3,397
Pseudo *R*^2^	0.010	0.033	0.009	0.038

*** *and*

** *respectively indicates significance at the 1 and 5% level; Robust standard errors are reported in parentheses*.

In addition, the control variables' results, such as gender, education, and local social support, contribute positively and significantly to the mental health condition of rural elderly adults, which is consistent with the research conclusions of Zhuori et al. (57). However, the distance between villages and towns has a negative and statistically significant influence on the mental health of rural elderly adults. The possible reasons are: the more remote the villages are, the more backward their pension infrastructure and public services are. The other variables were not significant.

Table 3 reports the estimated results of the impact of rural elderly adults' participation in the different types of local non-agricultural employment on their mental health. Models 5 and 7 are the regression results of the core explanatory variables to the dependent variable, which does not include the control variables. Models 6 and 8 add a series of control variables based on models 5 and 7. From the regression results in Table 3, the estimated coefficients of employed and self-employed variables in the mental health dimension are both positive, and both reject the null hypothesis at the statistical level of 1%. After verifying a series of control variables, the results show that rural elderly adults who participate in local employed work or self-employed work have better mental health compared with farming. Moreover, through the comparison of regression coefficients, it is found that the influence of being self-employed on mental health level is significantly greater than that of being employed, hence the hypothesis 2 is supported.

**Table 3:** Basic regression results: different types of non-agricultural employment.

**Variable name**	**Mental health**			
	**Model 5 (OLS)**	**Model 6 (OLS)**	**Model 7 (Ologit)**	**Model 8 (Ologit)**
Employed (Employee = 1;	0.144^***^	0.106^***^	0.663^***^	0.492^***^
Farming = 0)	(0.03)	(0.03)	(0.14)	(0.15)
Control variables	No	Yes	No	Yes
Observations	3,160	3,160	3,160	3,160
Pseudo *R*^2^	0.006	0.032	0.006	0.026
Self-employed (Self-employed/ Employer = 1; Farming = 0)	0.169^***^	0.144^**^	0.860^***^	0.759^***^
	(0.03)	(0.03)	(0.21)	(0.21)
Control variables	No	Yes	No	Yes
Observations	2,949	2,949	2,949	2,949
Pseudo R^2^	0.005	0.029	0.005	0.025

^***^ and ^**^ respectively indicates significance at the 1 and 5% level; Robust standard errors are reported in parentheses.

### Endogenous Analysis

Table 4 reports the two-stage least squares (2SLS) regression results after using the instrumental variable. Models 9–11 are the regression results of the first stage. From the results, the regression coefficients of instrumental variables to the three potential endogenous explanatory variables are all positive, rejecting the null hypothesis at the significance level of 1%, indicating instrumental variable with a strong correlation with endogenous explanatory variables. That is to say, the local non-agricultural economy can significantly increase the probability of rural elderly adults' participation in local non-agricultural employment (including employed and self-employed work). According to Stock and Yogo (58), if the statistical value of F in the first stage regression is >10, the original hypothesis of “there are weak instrumental variables” can be rejected, and the problem of weak instrumental variables is out of concern. Table 4 shows all the regression results of the statistical value of F in the first stage are >10, dismissing the possibility of weak instrumental variables. Models 12–14 are the second stage regression results. From the results, the coefficients of the three potential endogenous explanatory variables are significant at the statistical level of 1%, proving that participation in local non-agricultural employment (including employed and self-employed work) can significantly improve the mental health of rural elderly adults. This fact is consistent with the estimation results of benchmark model 1, determining the positive effect

**Table 4:** Instrumental variable method: 2SLS.

**Part A: The first stage**				**Part B: The second stage**		
	**Model 9 (Non-agricultural employment)**	**Model 10 (Employed)**	**Model 11 (Self-employed)**	**Model 12 (Mental health)**	**Model 13 (Mental health)**	**Model 14 (Mental health)**
Local non-agricultural economy	0.241^***^ (0.02)	0.192^***^ (0.02)	0.141^***^ (0.02)			
Non-agricultural employment				0.390^***^ (0.10)		
Employed					0.469^***^ (0.14)	
Self-employed						0.577^***^ (0.20)
Control variables	Yes	Yes	Yes	Yes	Yes	Yes
Observations	3,364	3,132	2,920	3,364	3,132	2,920
F statistics	47.11	27.07	14.00			
*R* ^2^	0.151	0.114	0.086	0.007	–	–

*** *indicates significance at the 1% level; Robust standard errors are reported in parentheses*.

of participation in local non-agricultural employment over the health of rural elderly adults.

### Robustness Check

This study uses the Logit model to estimate the score of rural elderly adults' tendency to participate in local non-agricultural employment (Table 5). The regression results show that gender, age, education, previous health condition, new rural insurance, the distance between villages and towns, local social support, local non-agricultural economy condition, rural traffic condition, and the local salary level significantly affects the rural elderly adults' participation in local non-agricultural employment, which is consistent with previous research results (59–61). In general terms, the proposed model can predict the probability of rural elderly adults' participation in local non-agricultural employment.

**Table 5:** Propensity score estimation based on Logit model.

**Variable**	**Coefficient**	**Robust standard error**
Gender (Male = 1; Female = 0)	0.839^***^	0.11
Age (Actual age)	−0.080^***^	0.01
Marriage (Married = 1; Others = 0)	−0.341	0.21
Education (Ranging from 1 = primary school to 4=undergraduate and above)	0.146^**^	0.07
Previous health condition (Good = 1; Bad= 0)	−0.278^*^	0.16
New rural insurance (Yes = 1; No = 0)	−0.376^***^	0.10
Distance between villages and towns (Actual distance)	−0.035^***^	0.01
Local social support (Numbers of local people who can provide support)	0.004^*^	0.00
Local non-agricultural economy condition (Yes = 1; No = 0)	1.171^***^	0.10
Rural traffic condition (Good = 1; Not good = 0)	0.733^***^	0.10
Local salary level (Daily wage)	0.007^***^	0.00
Log likelihood	−1373.059	
*R* ^2^	0.175	
LR chi2	583.10	
Observations	3,341	

^***^, ^**^, and ^*^ respectively indicates significance at the 1, 5, and 10% level.

**Figure 1:**
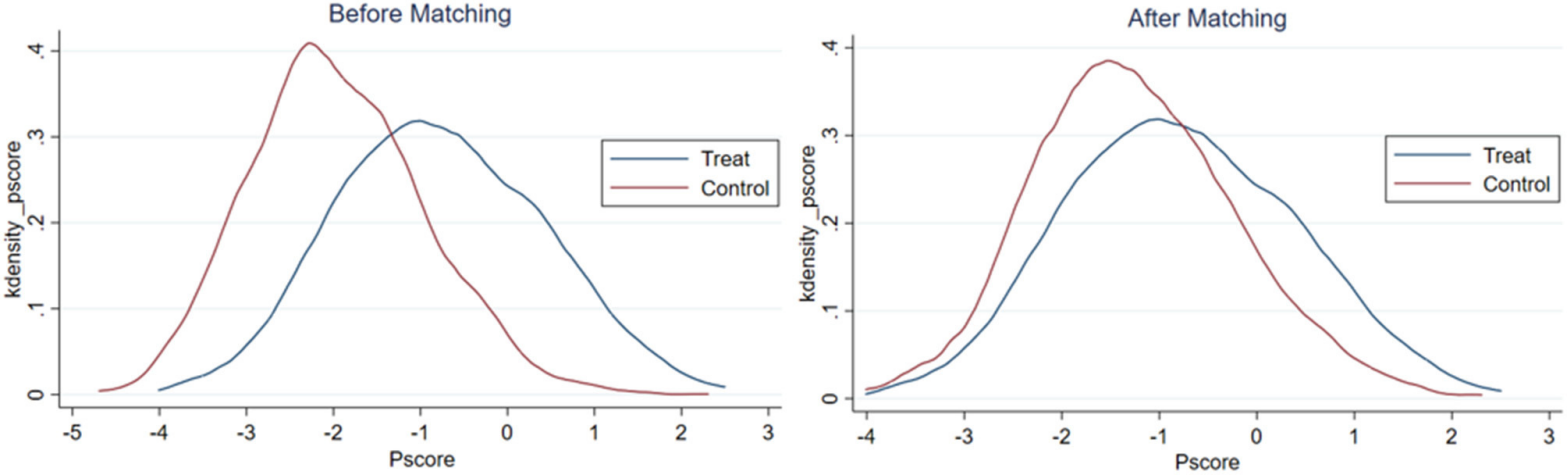
Results of balance hypothesis test: non-agricultural employment as a whole.

**Figure 2:**
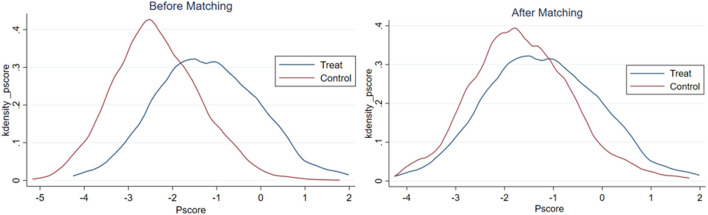
Results of balance hypothesis test: employed.

**Figure 3:**
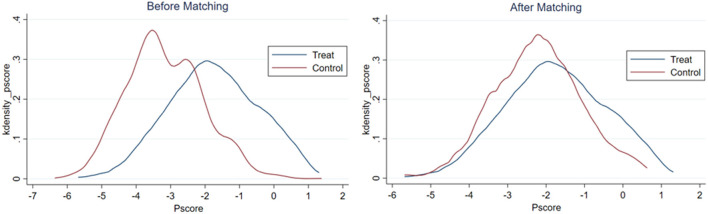
Results of balance hypothesis test: self-employed.

After estimating the propensity score of rural elderly adults to participate in local non-agricultural employment, this study matches the observed values within the common range to ensure the balance of propensity score matching. The results of the balance hypothesis test are reported in Figures 1–3. Before matching, there was a specific difference and peak deviation between the control group and the treatment group. After matching, the difference and peak deviation were significantly alleviated, and the standardized deviation of most variables after suiting is <10%, indicating that the matching effect is good (62).

After the balance hypothesis test, the ATT was estimated under different matching methods. This paper adopts three common matching methods: (1) K-nearest neighbor matching. In order to minimize the mean square error, K is set to 4. (2) Caliper matching. After optimal calculation, the caliper range is set to 0.07. (3) Kernel function matching. This paper uses the default kernel function and bandwidth. Table 6 shows the regression coefficient's consistent sign performance and significance on three analyzed methods, indicating a relatively robust treatment effect. These results are compatible with the regression results of benchmark model 1, proving that the estimation results in Tables 2, 3 are relatively robust to a certain extent.

**Table 6:** Robustness test: propensity score matching estimation results.

**Variable**	**Matching methods**	**Mental health**		
		**ATT**	**S.E**.	***T*-value**
Non-agricultural	K-nearest neighbor matching (k = 4)	0.092^***^	0.034	2.71
employment	Caliper matching (radius = 0.07)	0.096^***^	0.030	3.21
	Kernel function matching	0.096^***^	0.031	3.13
Employed	K-nearest neighbor matching (k = 4)	0.072^*^	0.038	1.90
	Caliper matching (radius = 0.07)	0.090^***^	0.032	2.79
	Kernel function matching	0.084^**^	0.033	2.56
Self-employed	K-nearest neighbor matching (k = 4)	0.115^**^	0.049	2.37
	Caliper matching (radius = 0.07)	0.121^***^	0.041	2.93
	Kernel function matching	0.122^***^	0.042	2.89

^***^, ^**^, and ^*^ respectively indicates significance at the 1, 5, and 10% level.

### Mechanism Analysis

The results of the above empirical analysis show that rural elderly adults' participation in local non-agricultural employment, including its different types, has significant positive effects on mental health. This section will continue to investigate the mechanism through which the rural elderly adults' participation in local non-agricultural employment has an impact on their mental health.

Table 7 reports the core independent variable impact on mediating variables' estimated results using the OLS and Ologit model. From the results of models 15–20, the estimated coefficients of the three core independent variables under the three mediating variables are all positive, and they all pass the significance level tests of 1 and 5%. It shows that the participation of rural elderly adults in local non-agricultural employment can significantly improve the income level, meet the needs of belongingness and respect, and develop human capital.

**Table 7:** Estimation results of independent variables to intermediary variables.

	**Income effect**		**Needs of belongingness and respect**		**Human capital development**	
	**Model 15 (OLS)**	**Model 16 (Ologit)**	**Model 17 (OLS)**	**Model 18 (Ologit)**	**Model 19 (OLS)**	**Model 20 (Ologit)**
Non-agricultural employment	0.229^***^ (0.05)	0.404^***^ (0.08)	0.207^***^ (0.04)	0.511^***^ (0.09)	0.184^***^ (0.04)	0.398^***^ (0.09)
Control variables	Yes	Yes	Yes	Yes	Yes	Yes
Observations	3,332	3,332	2,491	2,491	2,829	2,829
Pseudo *R*^2^	0.031	0.011	0.022	0.010	0.019	0.008
Employed	0.259^***^ (0.05)	0.452^***^ (0.095)	0.191^***^ (0.05)	0.483^***^ (0.11)	0.157^***^ (0.05)	0.336^***^ (0.10)
Control variables	Yes	Yes	Yes	Yes	Yes	Yes
Observations	3,098	3,098	2,319	2,319	2,620	2,620
Pseudo *R*^2^	0.033	0.012	0.018	0.009	0.014	0.006
Self-employed	0.162^**^ (0.08)	0.292^**^ (0.14)	0.221^***^ (0.06)	0.509^***^ (0.15)	0.220^***^ (0.06)	0.472^***^ (0.13)
Control variables	Yes	Yes	Yes	Yes	Yes	Yes
Observations	2,886	2,886	2,083	2,083	2,430	2,430
Pseudo *R*^2^	0.031	0.011	0.024	0.010	0.020	0.008

^***^ and ^**^ respectively indicates significance at the 1 and 5% level; Robust standard errors are reported in parentheses.

Table 8 reports the regression results after adding both explanatory variables and mediating variables. Models 21 to 26 estimated the mediation effects of incomes effect, needs of belongingness and respect, and human capital development, respectively. From the results of models 21–26, after adding mediating variables, local non-agricultural employment, needs of belongingness and respect, income effect, and human capital development were statistically significant, indicating that the mediating effect exists under these paths. Confirming once again the improvement on the mental health of rural elderly adults participating in local non-agricultural employment, through the satisfaction of belongingness and respect, the improvement of income, and the exertion of human capital. Hypotheses 3a to 3c are supported. Moreover, after adding the mediating variables, the coefficients of self-employed and employed variables under the three mediating variables are all positive and pass the significance test, which shows that both employed and self-employed non-agricultural work can improve their mental health through the satisfaction of belongingness and respect, the improvement of income and the exertion of human capital, it further proves the reliability of this research.

**Table 8:** Estimation results after adding independent variables and intermediary variables.

	**Mental health**		**Mental health**		**Mental health**		**Mediating effect**
	**Model 21 (OLS)**	**Model 22 (Ologit)**	**Model 23 (OLS)**	**Model 24 (Ologit)**	**Model 25 (OLS)**	**Model 26 (Ologit)**	
Non-agricultural employment	0.106^***^ (0.02)	0.540^***^ (0.13)	0.112^***^ (0.03)	0.568^***^ (0.14)	0.095^***^ (0.01)	0.504^***^ (0.14)	
Income effect	0.078^***^ (0.01)	0.346^***^ (0.04)					Existence
Needs of belongingness and respect			0.044^***^ (0.02)	0.266^***^ (0.06)			Existence
Human capital development					0.094^***^ (0.01)	0.410^***^ (0.06)	Existence
Control variables	Yes	Yes	Yes	Yes	Yes	Yes	
Observations	3,332	3,332	2,491	2,491	2,829	2,829	
Pseudo *R*^2^	0.052	0.045	0.036	0.037	0.054	0.045	
Employed	0.090^***^ (0.03)	0.448^***^ (0.15)	0.105^***^ (0.03)	0.494^***^ (0.16)	0.086^***^ (0.03)	0.438^***^ (0.16)	
Income effect	0.080^***^ (0.01)	0.344^***^ (0.04)					Existence
Needs of belongingness and respect			0.043^**^ (0.02)	0.254^***^ (0.06)			Existence
Human capital development					0.094^***^ (0.02)	0.406^***^ (0.06)	Existence
Control variables	Yes	Yes	Yes	Yes	Yes	Yes	
Observations	3,098	3,098	2,319	2,319	2,620	2,620	
Pseudo *R*^2^	0.051	0.042	0.036	0.035	0.054	0.043	
Self-employed	0.128^***^ (0.03)	0.699^***^ (0.21)	0.122^***^ (0.04)	0.732^***^ (0.26)	0.105^***^ (0.04)	0.604^***^ (0.23)	
Income effect	0.086^***^ (0.01)	0.361^***^ (0.04)					Existence
Needs of belongingness and respect			0.048^**^ (0.02)	0.278^***^ (0.07)			Existence
Human capital development					0.103^***^ (0.02)	0.423^***^ (0.06)	Existence
Control variables	Yes	Yes	Yes	Yes	Yes	Yes	
Observations	2,886	2,886	2,083	2,083	2,430	2,430	
Pseudo *R*^2^	0.050	0.043	0.031	0.034	0.053	0.043	

^***^ and ^**^ respectively indicates significance at the 1 and 5% level; Robust standard errors are reported in parentheses.

The findings from the study add fresh evidence to our understanding of the mental health implications associated with employment for rural elderly adults, including those in the middle-aged and even the pensioners in the rural non-agricultural economy. Our findings offer more nuanced explanations of the direct and indirect pathways through which the rural elderly who participate in local non-agricultural employment can improve their mental health. In the case of the direct effect pathways, the results from the study provides a substantial empirical evidence that, self-employment in the local non-agricultural activities in particular contributes more significant improvement to the mental health of the Elderly than wage employment.

Consistent with extant literature and in the context of this finding, the elderly adults feel more comfortable and enjoy life satisfaction when they work as self-employed than participating in the waged employment. It is obvious that during their retirement period, most of them may lack updated skills and abilities and would become less productive. In this instance, they cannot compete with the young and energetic Labor force particularly in the wage employment environment. Therefore, working as self-employed helps the elderly adults to work at their own pace while maintaining their satisfaction, well-being, and social connections.

Findings from the hypothesized relationships demonstrate that satisfaction of belongingness and respect, the improvement of income, and the exertion of human capital also have positive direct influence on the mental health of the elderly who participate in the local non-agricultural employment. The plausible explanation is that, the elderly adults who are employed or participate in self-employment are able to improve their income levels, ease any financial burden, and improve their life satiation, well-being, and mental stress, which are all indicators of a sound mental health (31–34). Again the continuous utilization of the physical strength, human capital and professional skills of the elderly contribute significantly to their mental health. This supports the assertion by previous studies such as (9) that utilizing human capital contributes to life expectancy, physical and mental health.

The empirical evidence from the mediation analysis offers another different perspectives on how local non-agriculture employment improves the mental health of the elderly through the satisfaction of belongingness and respect, the improvement of income, and the exertion of human capital development. Similarly, the findings contribute to the understudying of how the rural elderly who are into waged employment and self-employment in the local non-agricultural work can positively improve their mental health through channels such as the satisfaction of belongingness and respect, the improvement of income, and the exertion of human capital of the elderly adults.

## Conclusions and Policy Recommendations

In the context of the increasing situation of rural aging in China, the pension and health problems of rural elderly adults have become a significant policy concern. Based on the data from China Labor-force Dynamic Survey in 2016, this study analyzes the impact of rural elderly adults' participation in local non-agricultural employment on their mental health and its response mechanism.

In line with the findings of this study, we conclude that, the participation of rural elderly adults in local non-agricultural employment, including employed and self-employed work, significantly improves their mental health. Self-employment tends to have a greater positive contribution to the mental health of the elderly population than waged employment. Moreover, in terms of the specific impact mechanism, participation in local non-agricultural employment can improve the ability of the rural elderly adults to earn more income through their work, meet the needs of belongingness and respect, develop human capital, and then promote the improvement of mental health level.

Based on the above conclusion, this study makes feasible recommendations for the government, policymakers, and stakeholders to increase the investment in infrastructure and public services in rural areas while encouraging people and all kinds of organizations to bring capital and technology to start businesses in rural areas. There should be a promotion of the integration and development of rural industries that will gradually realize the prosperity of rural industries, and create more non-agricultural jobs for rural areas.

Again, the various pension schemes, NGOs, and the government should aim at actively developing the human resources, providing skills and vocational training for the rural left-behind elderly population. The rural elderly adults should be motivated and encouraged to participate in at least less stressful non-agricultural employment and entrepreneurship. Similarly, the government and the non-governmental organizations can explore more new mechanisms to foster rural employment and entrepreneurship for the elderly population in the rural areas.

Further, the rural social security system and the rural elderly population security level should be improved. The government should also formulate and improve relevant non-discriminatory policies and regulations to ensure the participation of the rural elderly adults in the process of employment, sharing a fair salary as young people, and improving the happiness level.

Moreover, based on the findings from the present study, we recommend that, the government of China and other Public Health-Based NGOs should develop a Comprehensive public health management programs for the rural elderly adults who seem to be left behind in the rural areas and feel lonely. This program can be implemented in cost effective approach such that, the online technology based medium can be used to reach out to the rural elderly particularly through mobile phone technology. Similarly, the public health institutions in the country should develop an intervention program where the public health officers will embark on home visitation to help the older adults to overcome their mental problems and psychological issues which at time push them into committing suicide. These programs can be used to improve the mental health and other psychological stress of the rural elderly adults in the rural China. The program will also further improve the well-being of the elderly adults in the rural China.

## Data Availability Statement

The original contributions presented in the study are included in the article/supplementary material, further inquiries can be directed to the corresponding author/s.

## Author Contributions

PJ: conceptualization, methodology, and writing original draft preparation. JZ: funding acquisition and supervision. AV: visualization and software. CO: writing reviewing and editing. JL: data curation and data collection. All authors contributed to the article and approved the submitted version.

## Funding

This research was funded by the National Social Science Foundation of China (18BRK003).

## Conflict of Interest

The authors declare that the research was conducted in the absence of any commercial or financial relationships that could be construed as a potential conflict of interest.

## Publisher's Note

All claims expressed in this article are solely those of the authors and do not necessarily represent those of their affiliated organizations, or those of the publisher, the editors and the reviewers. Any product that may be evaluated in this article, or claim that may be made by its manufacturer, is not guaranteed or endorsed by the publisher.
